# Mental distress among young adults in Great Britain: long-term trends and early changes during the COVID-19 pandemic

**DOI:** 10.1007/s00127-021-02194-7

**Published:** 2021-11-11

**Authors:** T. Gagné, I. Schoon, A. McMunn, A. Sacker

**Affiliations:** 1 International Centre for Lifecourse Studies in Society and Health, London, UK; 2 Department of Epidemiology and Public Health, University College London, 1-19 Torrington Place, office 536, London, WC1E 6BT UK; 3 Social Research Institute, UCL, London, UK

**Keywords:** United Kingdom, Young adults, Psychological distress, Time trends, General health questionnaire

## Abstract

**Purpose:**

In Great Britain, few studies documented mental health trends in young adults in the years preceding 2020, the mental health dimensions affected, and how these compare with changes observed during the COVID-19 pandemic.

**Methods:**

Long-term trends in mental health among 16–34 year old men and women between 1991 and 2018, and changes between 2018–19 and July–September 2020 were examined using all waves from the British Household Panel Study (1991–2008), the UK Household Longitudinal Study (2009–20), and the first five UKHLS COVID-19 waves administered in April, May, June, July, and September 2020. Findings are based on the GHQ-12 continuous score (0–36), clinically significant cases (4 + /12) and severe cases (7 + /12) for mental distress, and item endorsements.

**Results:**

Between 1991 and 2018, the prevalence of cases (4 + /12) increased from 14–22% to 19–32% across groups. Increases were largest in women aged 16–24. In April 2020, the risk of caseness (4 + /12) increased across groups by 55% to 80% compared to the 2018–19 baseline. This increase, however, rapidly diminished over time: in July–September 2020, there was only a higher risk of caseness (4 + /12) in men aged 25–34 (prevalence ratio = 1.29, 95% CI 1.01–1.65) compared to the 2018–19 baseline.

**Conclusion:**

Whereas distress surged in April 2020, its return to pre-pandemic levels by September 2020 highlights the nuanced impact that the pandemic may have over time. Given the magnitude of the decline in mental health over the past decade, attention must be given to young adults once the pandemic ends.

**Supplementary Information:**

The online version contains supplementary material available at 10.1007/s00127-021-02194-7.

## Introduction

Studies have documented worrisome trends in wellbeing and mental health disorders among young adults in past decades, with changes potentially accelerating over time [1–11]. This study explores the extent to which raised levels of mental distress during the first COVID-19 wave in Great Britain were an acceleration of these pre-existing trends. The focus is on young adults aged 16–34 given that increases in mental health problems in 2020 have been most significant for this age group [12–14]. This study distinguishes between those aged 16–24 and 25–34 as they are reaching different milestones across education, work and family life, and are likely to have had different responses to social change over time and recent changes in 2020 [15].

Relatively few studies in the United Kingdom have reported trends in mental health among young adults in the years preceding 2020. One study estimated trends in psychological distress among those aged 16–24 between 1991–2008 in Great Britain and found that there was a “polarization” of mental health in young women, with an increasing proportion reporting very high levels of distress during this period [16]. Another study examined trends between 1995–2014 among young people aged 4–24 across UK countries and found that long-standing mental health conditions increased most among those aged 16–24, from 1 to 6% between 1995–2014 in England [3]. Another UK report found that symptoms of depression and anxiety increased by 20% in women aged 16–24 between 2009–10 and 2011–12, and had not decreased in 2014–15 [17]. Finally, two other British studies reporting trends based on administrative data from primary care settings found that anxiety symptoms, diagnoses, and prescriptions among young adults had been stable since the late 1990s, started increasing around 2008, grew at a steeper rate around 2012, and showed no signs of deceleration up to 2018 [18, 19].

Contrasting with the smaller body of studies that investigated these long-term trends, the COVID-19 pandemic ignited a new wave of interest in monitoring mental health, including among young adults [12–14, 20, 21]. A study of the UK Household Longitudinal Study (UKHLS) COVID-19 survey found that levels of mental distress in April 2020 were 44% greater than pre-pandemic estimates, with larger increases found in those aged 16–24 and 25–34 [13]. Others found that distress levels recorded at the start of the first lockdown had likely improved in later months but remained high compared with pre-pandemic estimates, even when taking seasonality into account [14, 22]. Not considering disruptions in services for those with mental health issues, the pandemic and the government’s public health response created a range of unique stressors to young adults’ mental health, including fear of infection, pressure to cope with the lockdown, and uncertainty around its duration and efficacy [20, 21]. It also impacted many of the social and economic resources supporting young adults’ mental health in everyday life such as their financial security, employment and work conditions, social networks, and family life [23–26].

This paper thus aims to examine long-term trends and recent changes across mental health dimensions in the British young adult population, allowing us to compare the magnitude of differences over time and put 2020 in historical perspective. Doing so, we expand on the work of Banks and Xu [12] and others who analyzed the effects of the first months of the lockdown on mental distress using UKHLS data and drew attention to the different mental health dimensions measured with the GHQ-12 when studying changes across social contexts [12, 27]. Those studies found that: (1) increases in mental distress in April 2020 had been driven by the higher proportion reporting being unable to enjoy normal day-to-day activities, and (2) larger increases in young adults compared with older age groups had been driven by the higher proportions reporting feeling unhappy or depressed and thinking of themselves as worthless. Increases in mental distress in 2020 may be related to a narrower range of dimensions, such as a lower sense of control in response to the pandemic, whereas longer-term trends may relate to a wider range of dimensions following broader social and economic changes.

### Objectives

There is a need to pull existing threads together with a clear and precise focus on trends in young adult men and women using a nuanced understanding of the dimensionality of mental health. The goal of this study is to question how changes in mental distress among British young adults in 2020 compare with historical trends, and highlight the mental health burden that this age group was already facing before the pandemic started. The objectives are two-fold. The first is to estimate long-term trends between 1991–2018 and changes related to the pandemic between 2018–2020 in mental distress among British men and women aged 16–24 and 25–34, building on repeated surveys with large samples of young adults and a common measure of mental health assessed over time. The second is to disentangle differences over time by comparing changes across different mental health dimensions.

## Methods

### Data

To study changes in mental health before and during the COVID-19 pandemic, we used the 18 main waves from the British Household Panel Study (BHPS, 1991 to 2008), the 10 main waves from the UKHLS (2009–10 to 2018–19), and the first five COVID-19 main waves completed in April, May, June, July, and September 2020.

BHPS and UKHLS are household panel studies designed to provide high-quality longitudinal data to understand the long-term effects of social change and interventions on the wellbeing of the UK population. BHPS was an annually repeated panel of members aged 16 + from 5500 British households started in 1991 and continued until 2008. UKHLS started in 2009 using a similar design, and also started following BHPS members in 2010, representing at that point 38,313 households.

In 2020, UKHLS asked 42,330 respondents to complete a special survey to cover the impact of the pandemic in April, May, June, and July. In September, cohort members who participated in at least one previous COVID-19 wave were re-invited to participate in a fifth wave.

BHPS and UKHLS provide weights to produce representative estimates of the UK population taking into account the study design, baseline non-response, and attrition. Response rates for COVID-19 waves among those with a valid observation in Wave 9 ranged from 48% in April to 38% in September. In comparison, response rates for the main Wave 10 among Wave 9 participants was 88%. Sample sizes are detailed in Supplementary Table 1.

### Measures

Mental health was measured using the 12-item General Health Questionnaire (GHQ-12), a screening tool for identifying non-psychotic and minor psychiatric disorders in the general population [28]. Introduced with “The next questions are about how you have been feeling over the last few weeks”, the twelve items have a Likert-type four-point response scale coded from 0 to 3 (see Table 1).

**Table 1:** GHQ-12 item labels

#	Label	Full item	Response categories (4-point likert scale)	
			Least distressed (0)	Most distressed (3)
1	Concentrate	Have you recently been able to concentrate on whatever you're doing?	Better than usual	Much less than usual
2	Sleep loss	Have you recently lost much sleep over worry?	Not at all	Much more than usual
3	Useful	Have you recently felt that you were playing a useful part in things?	More so than usual	Much less than usual
4	Decisions	Have you recently felt capable of making decisions about things?	More so than usual	Much less than usual
5	Under strain	Have you recently felt constantly under strain?	Not at all	Much more than usual
6	Overcoming	Have you recently felt you couldn’t overcome your difficulties?	Not at all	Much more than usual
7	Enjoying	Have you recently been able to enjoy your normal day-to-day activities?	More so than usual	Much less than usual
8	Problems	Have you recently been able to face up to problems?	More so than usual	Much less than usual
9	Depressed	Have you recently been feeling unhappy or depressed?	Not at all	Much more than usual
10	Confidence	Have you recently been losing confidence in yourself?	Not at all	Much more than usual
11	Self-worth	Have you recently been thinking of yourself as a worthless person?	Not at all	Much more than usual
12	Happiness	Have you recently been feeling reasonably happy, all things considered?	More so than usual	Much less than usual

We used three different measures to capture mental distress: (1) scores ranging from 0 to 36 based on the summation of items on the Likert scale; (2–3) cases of mental distress and severe mental distress, defined as scoring ≥ 4 and ≥ 7 out of 12 on the “caseness” scale (scoring 2 or 3 on the four-point 0–3 scale) [28, 29]. To explore differences in mental health dimensions over time, we also examined endorsement (i.e., scoring 2 or 3 on the item scale) on GHQ items. We did not consider sub-scales as there remains a debate regarding whether items correlate on: (1) meaning, into social dysfunction (1, 3, 4, 7, 8, and 12), anxiety (2, 5, 6, 9), and loss of confidence (10 and 11), or (2) wording, into positive (1, 3, 4, 7, 8, and 12) and negative items (2, 5, 6, 9, 10, and 11) [30]. The median proportion of missingness on the GHQ score was 2.8% across waves. There was an elevated proportion of missingness in April 2020 (16.6%), explained by the high proportion of participants who left the survey before its completion: 13% of observations had partial interviews in April compared with less than 3% in subsequent waves. The probability of having a partial interview in April 2020 among participants aged 16–34, however, was not associated with reporting mental distress (*p* = 0.179) or severe mental distress (*p* = 0.444) in UKHLS Wave 10 (2018–19).

### Statistical analyses

Analyses were performed in two stages. For long-term trends, we described GHQ scores and cases each year between 1991 and 2018 in men and women aged 16–24 and 25–34 using data from the 18 BHPS waves and 10 UKHLS main waves. We then tested absolute differences in GHQ scores and relative differences in GHQ cases and item endorsements between the two 1991 and 2018 samples using linear regression for continuous outcomes and Poisson regression for dichotomous outcomes [32]. We also tested whether absolute differences in change coefficients for GHQ scores (linear betas), relative differences in change coefficients for GHQ cases (prevalence ratios), and relative differences in change coefficients for item endorsements (prevalence ratios) over time varied between sex and age groups using seemingly unrelated estimation (SUEST) [33]. We defined year-based samples using planned contact date following UKHLS guidelines [34]. As fieldwork continues months after the planned contact date, year-based samples include a proportion of cohort members interviewed over the start of the next year (range 0.0–33.5%, median: 5.8%, see Supplementary Table 1). As UKHLS waves were designed over a two-year period, we note that the 2009 sample represented by the Wave 1 Year 1 may under-represent ethnic minorities designed to be over-sampled in Year 2.

For early changes during the pandemic, we first described GHQ scores and cases across groups at six time-points using data from UKHLS Wave 10 (2018–19) and the five COVID-19 waves (April-September 2020). To minimize the risk that Wave 10 participants were affected by the pandemic, we only used observations interviewed before January 1st, 2020 (96.1% of eligible sample). We then tested differences in GHQ scores, cases, and item endorsements comparing estimates between 2018–19 and the last two waves, July and September 2020, pooled together to improve model efficiency, using random-intercept linear models for continuous outcomes and Poisson models for dichotomous outcomes. Differences were tested using mixed-effects models to integrate the nested nature of observations. To better interpret changes across months in 2020, we reproduced analyses comparing (1) Wave 10 and April 2020 and (2) April 2020 and July–September 2020 in Supplementary Tables 5 and 6.

**Fig. 1:**
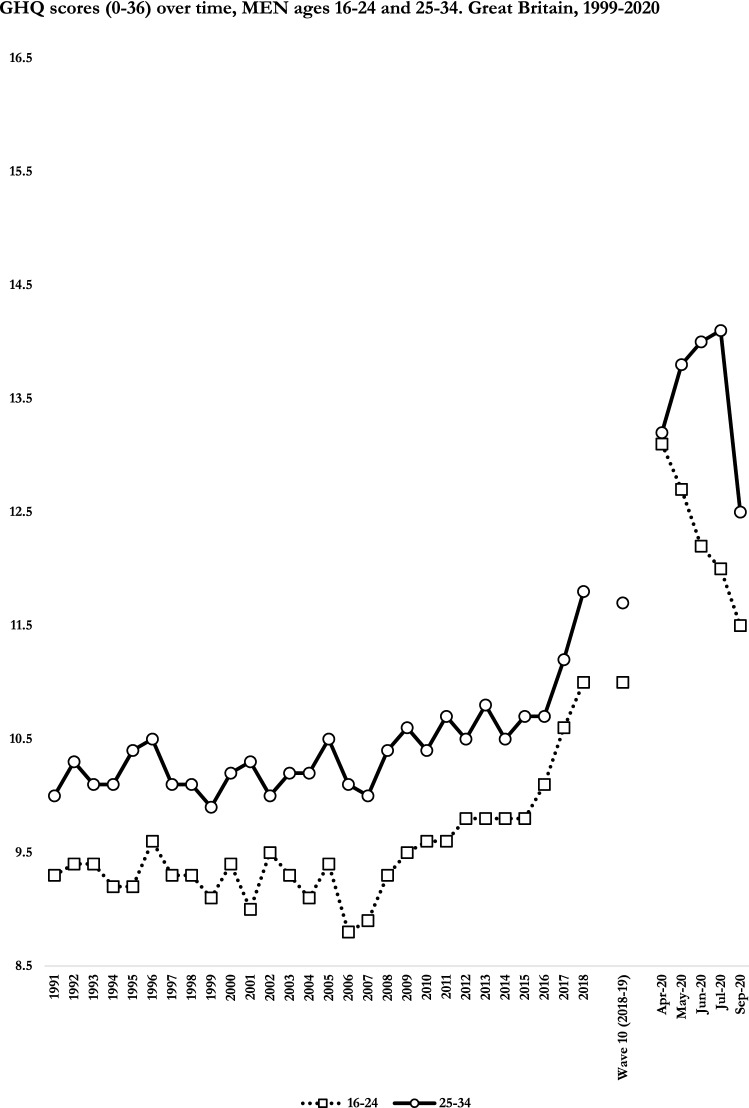
GHQ scores (0–36) over time, men ages 16–24 and 25–34. Great Britain, 1999–2020

**Fig. 2:**
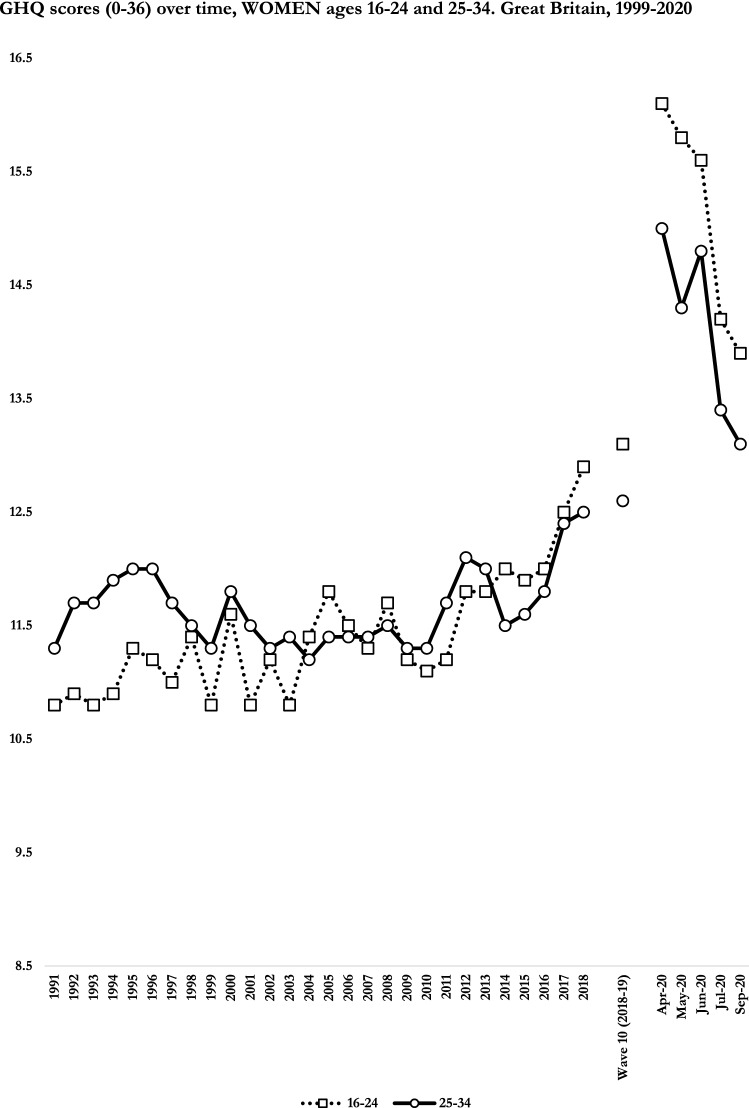
GHQ scores (0–36) over time, women ages 16–24 and 25–34. Great Britain, 1999–2020

We reported descriptive estimates in figures to facilitate interpretation, plotting GHQ scores in Figs. 1 and 2, and GHQ cases in the supplementary material (the full estimates are reported in Supplementary Tables 2–4). Estimates were adjusted for the UKHLS design variables and cross-sectional weights, and performed in “complete-case” samples using Stata 16 [31, 35].

## Results

### Long-term trends in mental distress at ages 16–34 in Great Britain, 1991–2018

Table 2 summarizes change coefficients, including 95% confidence intervals, for differences between 1991–2018.

**Table 2:** Long-term trends in mental distress, ages 16–34. Great Britain, BHPS/UKHLS. 1991–2018

Variable	16–24, M				16–24, F				25–34, M				25–34, F				SUEST
	1991	2018	Change	95%CI	1991	2018	Change	95%CI	1991	2018	Change	95%CI	1991	2018	Change	95%CI	*p*
Score (mean)	9.3	11.0	**1.68**	**1.18; 2.18**	10.8	12.9	**2.17**	**1.63; 2.71**	10.0	11.8	**1.77**	**1.20; 2.35**	11.3	12.5	**1.17**	**0.69; 1.66**	**0.042**
≥ 4 (%)	13.8	19.2	**1.39**	**1.10–1.77**	22.1	31.6	**1.43**	**1.21–1.70**	15.6	21.8	**1.40**	**1.14–1.72**	22.4	24.9	1.11	0.95–1.29	0.071
≥ 7 (%)	3.1	10.6	**3.37**	**2.12–5.36**	7.2	16.3	**2.26**	**1.63–3.13**	6.4	12.9	**2.03**	**1.48–2.77**	8.7	15.3	**1.75**	**1.37–2.24**	0.063
Concentrate (%)	14.7	16.9	1.15	0.90–1.47	18.3	24.6	**1.35**	**1.11–1.63**	14.9	20.3	**1.36**	**1.09–1.70**	22.9	22.1	0.97	0.82–1.14	**0.018**
Sleep loss (%)	11.9	11.3	0.95	0.72–1.26	21.0	23.0	1.09	0.91–1.32	14.6	18.1	1.24	0.98–1.57	20.0	19.6	0.98	0.83–1.17	0.369
Useful (%)	8.2	16.1	**1.96**	**1.43–2.67**	10.0	22.5	**2.25**	**1.74–2.91**	13.4	16.4	1.22	0.95–1.56	10.0	17.6	**1.77**	**1.39–2.25**	**0.010**
Decisions (%)	3.7	9.3	**2.48**	**1.58–3.90**	6.1	12.5	**2.04**	**1.48–2.80**	4.9	13.0	**2.64**	**1.89–3.68**	7.8	12.4	**1.59**	**1.19–2.12**	0.119
Under strain (%)	22.8	22.0	0.97	0.80–1.16	30.2	33.5	1.11	0.96–1.28	26.2	25.9	0.99	0.84–1.16	32.8	29.7	0.91	0.79–1.03	0.225
Overcoming (%)	9.0	17.1	**1.90**	**1.43–2.53**	15.3	25.1	**1.64**	**1.33–2.01**	10.3	18.0	**1.75**	**1.37–2.24**	15.3	17.1	1.12	0.92–1.36	**0.003**
Enjoying (%)	15.0	14.2	0.95	0.75–1.21	19.0	19.5	1.03	0.84–1.26	18.8	15.9	0.85	0.67–1.07	17.3	19.0	1.10	0.92–1.31	0.335
Problems (%)	5.1	11.6	**2.28**	**1.55–3.36**	10.6	15.0	**1.42**	**1.10–1.84**	6.5	13.3	**2.05**	**1.48–2.84**	11.5	12.9	1.12	0.89–1.41	**0.002**
Depressed (%)	19.8	21.6	1.09	0.90–1.32	30.1	31.5	1.05	0.91–1.21	17.5	23.6	**1.35**	**1.11–1.65**	27.7	25.7	0.93	0.80–1.08	**0.029**
Confidence (%)	9.1	16.6	**1.82**	**1.38–2.41**	15.7	31.5	**2.01**	**1.62–2.48**	10.5	19.1	**1.82**	**1.42–2.34**	11.9	25.5	**2.14**	**1.77–2.58**	0.698
Self-worth (%)	4.8	12.8	**2.65**	**1.84–3.83**	8.5	19.7	**2.31**	**1.74–3.05**	5.0	12.0	**2.41**	**1.70–3.44**	7.2	15.0	**2.10**	**1.61–2.74**	0.780
Happiness (%)	8.1	14.7	**1.81**	**1.35–2.42**	12.9	19.4	**1.50**	**1.19–1.89**	11.5	17.7	**1.54**	**1.22–1.96**	14.0	19.8	**1.41**	**1.16–1.71**	0.548

Change coefficients represent linear betas and prevalence ratios, weighted with clustered standard errors. The SUEST column reports the statistical significance of differences in change coefficients between groups. Bolded estimates are significant at the < .05 level. Differences across GHQ individual item change coefficients were significant in each group (*p* < .001)

In 1991, the prevalence of mental distress (GHQ 4 +) varied between 14% in men aged 16–24, 16% in men aged 25–34, 22% in women aged 16–24, and 22% in women aged 25–34. In 2018, mental distress significantly increased in all groups: 19% in men aged 16–24, 22% in men aged 25–34, 32% in women aged 16–24, and 25% in women aged 25–34. The prevalence of severe distress (GHQ 7 +) increased at a steeper rate, increasing from 1.75 times in women aged 25–34 (95% CI 1.37–2.24) to 3.37 times in men aged 16–24 (95% CI 2.12–5.36). Considering differences in trends across sexes and age groups, we found a larger increase in GHQ scores for women aged 16–24 (*b* = 2.17, 95% CI 1.63; 2.71) and a smaller increase among women aged 25–34 (*b* = 1.17, 95% CI 0.69; 1.66) between 1991–2018 (*p* = 0.042) (Table 2).

Whereas we did not formally test sub-trends between 1991–2018, we may distinguish three periods. First, between 1991 and 2006–10 there were no changes in distress among men, whereas there was an increase in 1991–95 followed by a decrease up to 2006–10 among women aged 25–34, and a steady increase across this period among women aged 16–24. Second, between 2006–10 and 2015–16, there was a pronounced increase across all groups that was steeper in women compared with men. Finally, in 2015–16, there was a second pronounced increase in distress affecting both sexes up to 2018.

Looking at item endorsements in Table 2, we observed significant differences in the magnitude of change across items in all groups (*p* < 0.001). Between 1991–2018, endorsements on three items did not change in all groups: “lost much sleep over worry”, “felt constantly under strain”, and “(not) able to enjoy your normal day-to-day activities”. There were significant increases: (1) on three items in all groups: “(not) capable of making decisions…”, “losing confidence in yourself”, and “thinking of yourself as a worthless person”; (2) on two other items in all groups except women aged 25–34, “couldn't overcome your difficulties” and “(not) able to face up to problems”; and 3) on a sixth item in all groups except women aged 16–24: “(not) playing a useful part in things”.

### Early changes during the pandemic in mental distress at ages 16–34 in Great Britain, 2018–2020

Table 3 summarizes change coefficients, including 95% confidence intervals, for differences across GHQ scores, cases, and item endorsements between the 2018–19 baseline and the pooled July–September 2020 sample.

**Table 3:** Early changes during the COVID pandemic in mental distress, ages 16–34. Great Britain, BHPS/UKHLS. 2018–2020

Variable	16–24, M				16–24, F				25–34, M				25–34, F			
	UW 10	CW 4–5	Change	95%CI	UW 10	CW 4–5	Change	95%CI	UW 10	CW 4–5	Change	95%CI	UW 10	CW 4–5	Change	95%CI
	2018–19	08/09–20			2018–19	08/09–20			2018–19	08/09–20			2018–19	08/09–20		
Score (mean)	11.0	11.8	**1.29**	**0.31; 2.27**	13.1	14.1	0.16	− 0.65; 0.97	11.7	13.3	**1.31**	**0.39; 2.23**	12.6	13.3	**1.00**	**0.38; 1.62**
≥ 4 (%)	20.2	17.3	0.85	0.58–1.24	32.1	31.9	0.88	0.73–1.06	21.8	28.0	**1.29**	**1.01–1.65**	24.7	25.3	1.05	0.88–1.25
≥ 7 (%)	10.7	11.9	1.23	0.75–2.01	17.5	20.5	1.04	0.79–1.38	13.6	18.1	1.21	0.89–1.66	15.4	15.1	1.08	0.84–1.39
Concentrate (%)	15.9	14.5	1.03	0.66–1.60	26.1	29.5	1.05	0.84–1.32	18.0	21.7	1.14	0.84–1.55	23.4	21.3	0.94	0.79–1.13
Sleep loss (%)	10.6	8.8	1.18	0.71–1.97	23.4	23.8	0.93	0.74–1.18	18.1	23.7	1.09	0.80–1.48	21.6	21.7	1.09	0.90–1.32
Useful (%)	17.0	17.2	1.08	0.70–1.68	23.9	28.0	1.01	0.81–1.27	17.3	19.4	0.98	0.74–1.30	16.9	19.3	1.10	0.87–1.40
Decisions (%)	9.7	11.8	1.44	0.88–2.35	14.1	18.8	1.26	0.96–1.67	12.5	13.1	1.14	0.79–1.65	10.9	14.8	**1.51**	**1.19–1.91**
Under strain (%)	20.4	17.3	0.71	0.50–1.01	32.8	31.4	0.89	0.73–1.08	24.0	33.1	1.24	1.00–1.55	30.8	30.3	1.00	0.86–1.17
Overcoming (%)	16.6	18.3	1.16	0.80–1.68	24.9	26.6	0.94	0.76–1.17	16.9	24.7	1.17	0.89–1.53	18.5	19.0	1.12	0.89–1.39
Enjoying (%)	14.7	17.8	1.34	0.87–2.06	19.3	24.8	1.21	0.95–1.53	16.9	24.3	**1.37**	**1.02–1.84**	18.5	24.0	**1.35**	**1.13–1.61**
Problems (%)	11.9	14.5	1.24	0.77–2.00	16.2	19.4	0.98	0.74–1.30	11.7	17.0	**1.57**	**1.11–2.22**	13.6	15.7	1.27	1.00–1.61
Depressed (%)	21.1	18.0	0.85	0.59–1.24	31.6	32.0	0.89	0.74–1.08	23.3	27.5	1.14	0.89–1.47	26.3	26.9	1.03	0.86–1.23
Confidence (%)	18.6	14.6	0.83	0.57–1.21	31.7	31.0	0.89	0.74–1.07	20.1	23.7	1.11	0.86–1.45	27.2	25.1	0.93	0.77–1.11
Self-worth (%)	12.7	11.7	0.99	0.57–1.73	20.1	20.0	0.85	0.67–1.09	13.0	15.4	1.14	0.85–1.54	14.6	16.1	1.18	0.92–1.52
Happiness (%)	13.7	13.5	1.07	0.68–1.69	19.8	20.6	0.99	0.76–1.29	17.3	24.4	**1.32**	**1.05–1.68**	18.2	20.7	1.15	0.92–1.44

Change coefficients are subject-specific linear betas and prevalence ratios from random-effects models, weighted with clustered standard errors. Bolded estimates are significant at the < .05 level

*UW 10* UKHLS wave 10 participants surveyed between January 2018 and December 2019, *CW 4–5* COVID waves 4–5 participants surveyed between July 24th and October 1st 2020

A similar trend was found across this period in all groups: we found high levels of distress in April 2020 that subsided over the following months. Compared with 2018–19 estimates, the risk of distress (GHQ 4 +) in April 2020 significantly increased at a similar rate across all groups, with PRs ranging from 1.55 (95% CI 1.25–1.92) in men aged 25–34 to 1.80 (95% CI 1.56–2.08) in women aged 25–34 (Supplementary Table 5)*.* Between April and July–September 2020, the risk of distress then significantly decreased all groups by 21% to 46% (Supplementary Table 6). Whereas the increases in April were similar across groups, the decreases in July–September were smaller in men aged 25–34 (PR = 0.79, 95% CI 0.65–0.97) compared with women aged 16–24 (PR = 0.54, 95% 0.45–0.65) and aged 25–34 (PR = 0.57, 95% CI 0.48–0.67). Comparing 2018–19 with July–September 2020, there were few differences in the risk of caseness across groups, with significant increases in distress only found in men aged 25–34 (PR = 1.29, 95% 1.01–1.65).

Regarding item endorsements, we only found differences between 2018–19 and July–September 2020 among men aged 25–34 for “(not) able to enjoy your normal day-to-day activities”, “(not) able to face up to problems”, and “(not) feeling reasonably happy, all things considered”, and among women aged 25–34 for “(not) able to enjoy your normal day-to-day activities” and “(not) capable of making decisions…”. In April 2020, the increase in GHQ scores was largely attributable to the increase in endorsements on “(not) able to enjoy your normal day-to-day activities”, “(not) capable of making decisions…”, and “(not) playing a useful part in things” (Supplementary Table 5).

## Discussion

In light of the crisis affecting British young adults in 2020–21 and the relative lack of evidence on long-term mental health trends [13, 14, 36], this paper sought to compare changes in mental health dimensions over the past three decades and during the COVID-19 pandemic. The high levels of mental distress over the first half of 2020 appear to have been the result of the continuation of pre-existing trends, marked by the Great Recession and the UK government austerity program, and an initial shock reaction in March–April to the COVID-19 pandemic and lockdown measures which then subsided [37]. Research has yet to unpack the underlying changes driving these trends, which may include increases in social media use and decreases in sleep quality, but also declining employment opportunities, reduced real income, and more precarious work conditions, as well as new living arrangements with parents, partners, and others [5, 6, 38]. There is also debate about the extent to which new generations enter the transition to adulthood in a more vulnerable state as mental health has also declined in younger age groups over time [3, 16]. Looking at annual trends since 1991, we found that mental health has declined substantially in all groups. Unlike previous studies suggesting that declines have been concentrated among young women, changes were marked for both men and women [16]. The prevalence of severe distress grew at an especially steep rate, doubling across groups and tripling in men aged 16–24 between 1991–2018.

Focusing on experiences during 2020, the findings support other studies, in that British young adults experienced historically high levels of distress at the height of the first lockdown. However, the levels of distress reported in April 2020 were followed by large decreases over the following months, resulting in relatively small differences between pre-pandemic levels and September 2020. We note that our analysis differs from earlier studies with the use of a more recent baseline (Wave 10 in 2018–19), which may have led to a more conservative estimate of changes attributable to the pandemic [39]. That is, the use of a baseline measured one year earlier (Wave 9 in 2017–18) would not capture any trend present between 2017–18 and 2018–19.

The findings suggest that many young adults may have had the capacity to adapt relatively rapidly to the first lockdown and return to levels observed in 2018–19. This finding is in line with evidence from disaster sciences, which argue that disasters tend to cause long-term psychological harm in a minority of exposed individuals [40]. We should however be cautious in generalizing these results over time: (1) changes between April and September may have included seasonal effects (i.e., summer months being associated with lower distress), and (2) July and September 2020 were months with relatively few COVID-19 cases and decreased public health restrictions. Unfortunately, the second and third COVID-19 waves that started after September 2020 has been far worse compared with the first one in terms of case and death rates, social and economic support from the British government, and length of time.

We can use changes observed between April and September 2020 to put long-term trends between 1991–2018 into perspective. Comparing differences in scores between 1991–2018 and 2018–2020, the 28 year increase in distress could be equated to 94–97% of the estimated impact of the first lockdown in men (*b*_1991-2018_ = 1.7 *versus b*_W10-Apr20_ = 1.7) and women (*b*_1991-2018_ = 2.2 *versus b*_W10-Apr20_ = 2.3) aged 16–24. The 1991–2018 increase was greater than the estimated impact of the pandemic in men aged 25–34 (*b*_1991-2018_ = 1.8 *versus b*_W10-Apr20_ = 1.2) and reached approximately 40% in women aged 25–34 (*b*_1991-2018_ = 1.2 *versus b*_W10-Apr20_ = 2.9). The findings regarding changes across item endorsements highlight that changes between 1991–2018 and during 2020 are not directly comparable as higher risks in April 2020 were driven in large part by young adults’ lower capacity to perform and enjoy day-to-day activities. The capacity to compare results for the analyses of long-term trends and early changes during the pandemic also depends on our ability to derive unbiased estimates, which ultimately requires the triangulation of different methods and datasets.

The findings shed new light on the magnitude of the mental health burden already present in the British young adult population in the years leading up to 2020. A key issue over the next years will be to better understand how the drivers of long-term trends and early changes during the pandemic intersect to shape mental health problems in this age group moving forward. Evidence from previous recessions suggest that the economic impacts of the pandemic on the mental health of young adults may only appear years after it started [41]. In the short-term, many of the determinants of mental health—employment and financial security, work and family-life balance, social contact and support, access to social and mental health services—have worsened in response to the lack of government support [12, 13, 42]. Whereas the UK government implemented a furlough scheme to buffer the effects of job loss, preliminary evidence suggests that it may have had limited benefits on mental distress [43]. Young adults may also have suffered more from public health restrictions and social isolation compared with other age groups [44, 45]. These determinants are likely to have multiplicative effects, meaning that more vulnerable groups may have been at high risk of developing mental health problems from the experience of accumulated disruptions over the course of the pandemic [23]. Building on the first group of studies that highlighted young parents and those recently unemployed to be at higher risk of distress during the start of the pandemic, research needs to better inform which groups have fared worst in the pandemic and what may be done for them in the short and long term [12, 13, 36, 46].

### Strengths and limitations

This study builds on the methodological strengths of the UKHLS to produce representative estimates of the British population between 1991–2020. Regarding limitations, we first highlight that long-term trends may not be solely attributable to period effects, and may also result from the change in composition of young adult populations over time. Future studies need to use robust statistical adjustment strategies to unpack the magnitude of this period effect beyond changes in employment, job conditions, housing, family life, etc. Second, we note that attrition across UKHLS has been higher among young adults, and that the COVID-19 survey waves have relatively low response rates and small young adult samples. Some estimates may therefore be under-powered and under-represent groups at higher risk of distress [47]. Finally, larger sample sizes would have enabled us to further stratify our samples into smaller age brackets to robustly capture the different transition stages during young adulthood (e.g., 16–19 and 20–24). Having access to other repeated measures beyond the GHQ-12 would have also enabled us to better capture the complex mental health burden experienced by young adults before and during the pandemic.

### Conclusion

This study offers robust evidence on trends in mental distress over the past thirty years, including during the first six months following the first COVID-19 lockdown, among British young adult men and women. Rising mental health problems among young adults were already observed before the pandemic. We highlighted the marked increase in distress that young adult generations have been facing over time, in particular over the past ten years. Going back to pre-pandemic levels of mental health should therefore not suffice as a public health target. The findings emphasize the need for systemic improvement to address the mental health crisis and a strong vision to promote the wellbeing of British young adults long after the pandemic ends. This strategy needs to be framed in light of the COVID-19 recession, which is likely to have deep scarring effects on the future of current young adult generations.

## Supplementary Information

Below is the link to the electronic supplementary material.

Supplementary file1 (DOCX 63 KB)

## Data Availability

The main data are publicly available via UK Data Service repository (study numbers 6614 and 8644).

## Code availability

The Stata code is available on request to the corresponding author.
