# Supplementary material for: Mental distress among young adults in Great Britain: long-term trends and early changes during the COVID-19 pandemic
Source: Soc Psychiatry Psychiatr Epidemiol. 2021 Nov 11;57(6):1261–72. doi: 10.1007/s00127-021-02194-7 (PMC8583581; doi:10.1007/s00127-021-02194-7)
Supplement: Supplementary file 1 — Supplementary file1 (DOCX 63 KB) [file 127_2021_2194_MOESM1_ESM.docx]

**SUPPLEMENTARY MATERIAL**

**Title**

Mental distress among young adults in Great Britain: long-term trends and early changes during the COVID pandemic

**Table of contents:**

- Table S1: Sample size
- Table S2: Numbers for Figures: mean GHQ score
- Table S3: Numbers for Figures: mental distress (≥4 GHQ-12)
- Table S4: Numbers for Figures: severe mental distress (≥7 GHQ-12)
- Table S5: Recent changes in GHQ: comparing Wave 10 (2018-19) and April 2020
- Table S6: Recent changes in GHQ: comparing April 2020 and July-September 2020
- Figure S1: Long-term trends for GHQ threshold scores
- Figure S2: Recent changes for GHQ threshold scores

**SUPPLEMENTARY TABLE 1**

**Sample sizes in the BHPS, UKHLS, and UKHLS COVID waves**

| **Year** | **Sample size (*n*)** | | | | **Interview date is in the year after the planned contact year (%)** |
| --- | --- | --- | --- | --- | --- |
|  | **16-24, M** | **16-24, F** | **25-34, M** | **25-34, F** |  |
|  | Valid *n* | Valid *n* | Valid *n* | Valid *n* |  |
|  |  |  |  |  |  |
| 1991 | 754 | 738 | 947 | 1,063 | 0.0 |
| 1992 | 735 | 749 | 912 | 1,051 | 3.0 |
| 1993 | 718 | 747 | 872 | 1,022 | 6.0 |
| 1994 | 731 | 739 | 865 | 1,007 | 5.4 |
| 1995 | 698 | 730 | 830 | 971 | 6.7 |
| 1996 | 733 | 762 | 863 | 985 | 3.4 |
| 1997 | 802 | 831 | 984 | 1,133 | 3.7 |
| 1998 | 735 | 796 | 940 | 1,104 | 4.0 |
| 1999 | 967 | 1,146 | 1,285 | 1,491 | 28.1 |
| 2000 | 988 | 1,115 | 1,228 | 1,407 | 33.5 |
| 2001 | 1,007 | 1,125 | 1,158 | 1,305 | 11.8 |
| 2002 | 846 | 931 | 994 | 1,133 | 12.2 |
| 2003 | 841 | 904 | 940 | 1,068 | 3.3 |
| 2004 | 789 | 866 | 918 | 1,037 | 4.7 |
| 2005 | 778 | 916 | 889 | 1,025 | 4.0 |
| 2006 | 784 | 898 | 858 | 984 | 3.7 |
| 2007 | 729 | 924 | 805 | 903 | 3.1 |
| 2008 | 715 | 843 | 741 | 870 | 2.4 |
| 2009 | 1,235 | 1,466 | 1,382 | 1,847 | 4.9 |
| 2010 | 2,002 | 2,433 | 2,188 | 3,100 | 7.3 |
| 2011 | 2,171 | 2,626 | 2,317 | 3,183 | 5.6 |
| 2012 | 1,999 | 2,326 | 1,998 | 2,789 | 6.3 |
| 2013 | 1,918 | 2,184 | 1,794 | 2,498 | 8.2 |
| 2014 | 1,839 | 2,057 | 1,678 | 2,286 | 7.6 |
| 2015 | 1,665 | 1,930 | 1,565 | 2,057 | 9.2 |
| 2016 | 1,809 | 2,025 | 1,630 | 2,210 | 9.5 |
| 2017 | 1,502 | 1,763 | 1,362 | 1,898 | 11.1 |
| 2018 | 1,273 | 1,533 | 1,165 | 1,605 | 9.5 |
|  |  |  |  |  |  |
| Wave 10 (2018-19) | 1,160 | 1,437 | 1,082 | 1,468 |  |
|  |  |  |  |  |  |
| April 2020 | 219 | 451 | 406 | 703 |  |
| May 2020 | 162 | 370 | 332 | 597 |  |
| June 2020 | 143 | 322 | 297 | 552 |  |
| July 2020 | 148 | 289 | 297 | 540 |  |
| Sept 2020 | 114 | 243 | 260 | 469 |  |
|  |  |  |  |  |  |

Valid *n* is defined as 1) being in England, Wales, or Scotland, and having valid data on: 2) overall GHQ score; 3) cross-sectional weight.

We note that Wave 10 numbers refer to the 96.1% of valid participants who answered before December 31^st^, 2019.

See BHPS User manual, p. A4-8, and UKHLS User guide November 2019, p. 16, for definition of cross-sectional weights.

<https://www.iser.essex.ac.uk/bhps/documentation/pdf_versions/volumes/5151userguide_vola.pdf>

<https://www.understandingsociety.ac.uk/sites/default/files/downloads/general/weighting_faqs.pdf>

**SUPPLEMENTARY TABLE 2**

**ESTIMATES FOR FIGURES**

**Mean GHQ-12 (0-36), young adults ages 16-34. BHPS/UKHLS, 1991-2020.**

|  | **16-24, M** | | **16-24, F** | | **25-34, M** | | **25-34, F** | |
| --- | --- | --- | --- | --- | --- | --- | --- | --- |
| **Year** | Mean | 95%CI | Mean | 95%CI | Mean | 95%CI | Mean | 95%CI |
|  |  |  |  |  |  |  |  |  |
| 1991 | 9.3 | 9.0-9.6 | 10.8 | 10.4-11.1 | 10.0 | 9.7-10.3 | 11.3 | 11.0-11.6 |
| 1992 | 9.4 | 9.0-9.7 | 10.9 | 10.5-11.3 | 10.3 | 10.0-10.7 | 11.7 | 11.3-12.0 |
| 1993 | 9.4 | 9.0-9.7 | 10.8 | 10.4-11.2 | 10.1 | 9.7-10.5 | 11.7 | 11.3-12.0 |
| 1994 | 9.2 | 8.8-9.6 | 10.9 | 10.4-11.4 | 10.1 | 9.7-10.4 | 11.9 | 11.6-12.3 |
| 1995 | 9.2 | 8.9-9.6 | 11.3 | 10.8-11.7 | 10.4 | 10.0-10.8 | 12.0 | 11.6-12.4 |
| 1996 | 9.6 | 9.2-10.0 | 11.2 | 10.7-11.8 | 10.5 | 10.1-10.9 | 12.0 | 11.5-12.4 |
| 1997 | 9.3 | 9.0-9.7 | 11.0 | 10.5-11.4 | 10.1 | 9.8-10.4 | 11.7 | 11.4-12.0 |
| 1998 | 9.3 | 8.9-9.7 | 11.4 | 11.0-11.9 | 10.1 | 9.7-10.5 | 11.5 | 11.2-11.9 |
| 1999 | 9.1 | 8.7-9.5 | 10.8 | 10.4-11.2 | 9.9 | 9.6-10.2 | 11.3 | 11.0-11.7 |
| 2000 | 9.4 | 9.0-9.7 | 11.6 | 11.1-12.0 | 10.2 | 9.8-10.5 | 11.8 | 11.4-12.2 |
| 2001 | 9.0 | 8.6-9.4 | 10.8 | 10.4-11.2 | 10.3 | 9.9-10.6 | 11.5 | 11.1-11.9 |
| 2002 | 9.5 | 9.1-9.9 | 11.2 | 10.8-11.7 | 10.0 | 9.6-10.4 | 11.3 | 10.9-11.8 |
| 2003 | 9.3 | 8.8-9.7 | 10.8 | 10.3-11.3 | 10.2 | 9.9-10.6 | 11.4 | 10.9-11.8 |
| 2004 | 9.1 | 8.7-9.5 | 11.4 | 10.9-11.9 | 10.2 | 9.8-10.6 | 11.2 | 10.7-11.6 |
| 2005 | 9.4 | 9.0-9.9 | 11.8 | 11.2-12.4 | 10.5 | 10.1-10.9 | 11.4 | 10.9-11.8 |
| 2006 | 8.8 | 8.4-9.3 | 11.5 | 11.0-12.1 | 10.1 | 9.7-10.5 | 11.4 | 10.9-11.9 |
| 2007 | 8.9 | 8.4-9.3 | 11.3 | 10.7-11.8 | 10.0 | 9.5-10.4 | 11.4 | 11.0-11.9 |
| 2008 | 9.3 | 8.8-9.8 | 11.7 | 11.0-12.3 | 10.4 | 9.9-10.9 | 11.5 | 11.0-12.0 |
| 2009 | 9.5 | 9.2-9.8 | 11.2 | 10.9-11.5 | 10.6 | 10.3-10.9 | 11.3 | 11.1-11.6 |
| 2010 | 9.6 | 9.4-9.9 | 11.1 | 10.8-11.3 | 10.4 | 10.2-10.7 | 11.3 | 11.1-11.5 |
| 2011 | 9.6 | 9.4-9.8 | 11.2 | 11.0-11.5 | 10.7 | 10.4-10.9 | 11.7 | 11.4-11.9 |
| 2012 | 9.8 | 9.6-10.1 | 11.8 | 11.5-12.1 | 10.5 | 10.2-10.8 | 12.1 | 11.8-12.4 |
| 2013 | 9.8 | 9.6-10.1 | 11.8 | 11.5-12.1 | 10.8 | 10.5-11.1 | 12.0 | 11.7-12.3 |
| 2014 | 9.8 | 9.6-10.1 | 12.0 | 11.7-12.4 | 10.5 | 10.2-10.8 | 11.5 | 11.2-11.7 |
| 2015 | 9.8 | 9.5-10.1 | 11.9 | 11.6-12.2 | 10.7 | 10.4-11.0 | 11.6 | 11.3-11.9 |
| 2016 | 10.1 | 9.8-10.4 | 12.0 | 11.7-12.3 | 10.7 | 10.4-11.1 | 11.8 | 11.5-12.1 |
| 2017 | 10.6 | 10.3-11.0 | 12.5 | 12.2-12.9 | 11.2 | 10.8-11.6 | 12.4 | 12.0-12.7 |
| 2018 | 11.0 | 10.6-11.4 | 12.9 | 12.5-13.3 | 11.8 | 11.3-12.3 | 12.5 | 12.1-12.9 |
|  |  |  |  |  |  |  |  |  |
| W10 (2018-19) | 11.0 | 10.6-11.4 | 13.1 | 12.7-13.6 | 11.7 | 11.3-12.2 | 12.6 | 12.1-13.0 |
| April 2020 | 13.1 | 11.8-14.3 | 16.1 | 15.3-16.8 | 13.2 | 12.4-14.1 | 15.0 | 14.2-15.9 |
| May 2020 | 12.7 | 11.6-13.7 | 15.8 | 14.7-16.9 | 13.8 | 12.8-14.8 | 14.3 | 13.4-15.2 |
| June 2020 | 12.2 | 10.9-13.4 | 15.6 | 14.5-16.8 | 14.0 | 12.9-15.1 | 14.8 | 13.3-16.3 |
| July 2020 | 12.0 | 10.6-13.5 | 14.2 | 13.3-15.1 | 14.1 | 12.8-15.4 | 13.4 | 12.5-14.2 |
| Sept 2020 | 11.5 | 10.0-13.0 | 13.9 | 13.0-14.9 | 12.5 | 11.5-13.5 | 13.1 | 12.1-14.1 |
|  |  |  |  |  |  |  |  |  |

**SUPPLEMENTARY TABLE 3**

**ESTIMATES FOR FIGURES**

**Mental distress (≥4 GHQ-12), ages 16-34. BHPS/UKHLS, 1991-2020.**

|  | **16-24, M** | | **16-24, F** | | **25-34, M** | | **25-34, F** | |
| --- | --- | --- | --- | --- | --- | --- | --- | --- |
| **Year** | % | 95%CI | % | 95%CI | % | 95%CI | % | 95%CI |
|  |  |  |  |  |  |  |  |  |
| 1991 | 13.8 | 11.2-16.8 | 22.1 | 19.1-25.4 | 15.6 | 13.4-18.0 | 22.4 | 20.2-24.9 |
| 1992 | 14.5 | 11.8-17.7 | 21.2 | 17.9-24.9 | 18.8 | 16.0-21.9 | 25.3 | 22.6-28.2 |
| 1993 | 15.5 | 12.7-18.9 | 22.9 | 19.6-26.5 | 16.5 | 13.8-19.7 | 22.9 | 20.0-26.0 |
| 1994 | 13.8 | 11.4-16.5 | 22.5 | 19.0-26.5 | 16.5 | 13.9-19.5 | 25.7 | 22.8-28.7 |
| 1995 | 12.1 | 9.5-15.2 | 25.4 | 21.7-29.5 | 18.0 | 15.1-21.3 | 23.4 | 20.9-26.2 |
| 1996 | 14.7 | 12.1-17.8 | 26.2 | 22.4-30.3 | 18.4 | 15.6-21.5 | 26.0 | 22.7-29.6 |
| 1997 | 14.2 | 11.5-17.3 | 23.7 | 20.8-26.9 | 16.0 | 13.9-18.3 | 24.5 | 21.8-27.4 |
| 1998 | 13.4 | 10.7-16.7 | 23.7 | 20.4-27.4 | 15.2 | 12.8-18.0 | 23.2 | 20.7-25.9 |
| 1999 | 12.5 | 10.0-15.5 | 21.0 | 18.0-24.4 | 14.0 | 11.8-16.6 | 20.6 | 18.0-23.4 |
| 2000 | 13.8 | 11.5-16.5 | 26.5 | 23.2-30.1 | 14.6 | 12.4-17.0 | 23.8 | 21.2-26.7 |
| 2001 | 11.5 | 9.2-14.3 | 23.4 | 20.5-26.6 | 16.6 | 14.1-19.5 | 24.8 | 22.1-27.7 |
| 2002 | 16.3 | 13.5-19.5 | 23.2 | 20.0-26.7 | 14.5 | 11.8-17.8 | 23.5 | 20.3-27.1 |
| 2003 | 14.4 | 11.9-17.3 | 24.0 | 20.4-28.1 | 16.4 | 13.9-19.2 | 21.8 | 18.8-25.1 |
| 2004 | 13.9 | 11.3-16.9 | 25.6 | 21.9-29.6 | 15.9 | 13.1-19.2 | 19.8 | 17.0-22.9 |
| 2005 | 16.7 | 13.7-20.1 | 28.2 | 24.5-32.2 | 17.3 | 14.4-20.6 | 22.9 | 19.9-26.3 |
| 2006 | 11.8 | 9.2-15.0 | 27.4 | 23.5-31.8 | 15.4 | 12.7-18.7 | 22.2 | 18.9-25.9 |
| 2007 | 14.1 | 11.4-17.3 | 24.4 | 20.8-28.3 | 16.6 | 13.5-20.3 | 22.1 | 18.8-25.8 |
| 2008 | 16.6 | 13.6-20.1 | 27.0 | 22.9-31.5 | 15.5 | 12.5-19.1 | 21.9 | 18.4-25.7 |
| 2009 | 14.1 | 12.2-16.2 | 21.9 | 19.6-24.4 | 17.6 | 15.5-19.8 | 21.2 | 19.2-23.3 |
| 2010 | 15.0 | 13.4-16.8 | 21.1 | 19.3-23.1 | 15.5 | 13.9-17.2 | 20.5 | 18.8-22.2 |
| 2011 | 13.9 | 12.3-15.6 | 22.2 | 20.4-24.1 | 17.1 | 15.4-19.0 | 21.5 | 19.8-23.2 |
| 2012 | 16.2 | 14.5-18.1 | 26.6 | 24.5-28.7 | 16.9 | 15.0-18.9 | 24.9 | 23.2-26.8 |
| 2013 | 16.0 | 14.2-18.0 | 26.1 | 24.0-28.2 | 17.4 | 15.4-19.6 | 24.6 | 22.6-26.6 |
| 2014 | 16.0 | 14.2-18.0 | 27.2 | 24.9-29.6 | 16.7 | 14.6-19.1 | 21.8 | 19.9-23.9 |
| 2015 | 14.7 | 12.9-16.7 | 26.3 | 24.0-28.8 | 18.0 | 15.7-20.6 | 22.3 | 20.2-24.6 |
| 2016 | 16.3 | 14.3-18.5 | 25.8 | 23.5-28.2 | 17.5 | 15.3-19.9 | 22.9 | 20.8-25.2 |
| 2017 | 18.0 | 15.8-20.5 | 29.1 | 26.6-31.8 | 19.9 | 17.4-22.7 | 25.9 | 23.3-28.5 |
| 2018 | 19.2 | 16.7-22.0 | 31.6 | 28.9-34.5 | 21.8 | 18.7-25.1 | 24.9 | 22.3-27.7 |
|  |  |  |  |  |  |  |  |  |
| W10 (2018-19) | 20.2 | 17.4-23.2 | 32.1 | 29.0-35.4 | 21.8 | 18.6-25.5 | 24.7 | 21.7-27.9 |
| April 2020 | 32.0 | 23.8-41.6 | 55.3 | 48.8-61.6 | 35.9 | 28.7-43.8 | 43.9 | 37.9-50.1 |
| May 2020 | 29.3 | 20.8-39.5 | 47.7 | 40.1-55.5 | 34.4 | 27.4-42.2 | 38.0 | 31.7-44.8 |
| June 2020 | 24.6 | 15.7-36.5 | 47.2 | 38.7-55.8 | 33.8 | 26.4-42.2 | 35.7 | 28.9-43.1 |
| July 2020 | 15.6 | 8.8-26.2 | 33.0 | 25.0-42.1 | 31.8 | 23.5-41.4 | 25.1 | 20.1-30.9 |
| Sept 2020 | 19.1 | 10.8-31.6 | 30.7 | 22.8-39.9 | 23.9 | 17.5-31.9 | 25.6 | 20.2-31.9 |
|  |  |  |  |  |  |  |  |  |

**SUPPLEMENTARY TABLE 4**

**ESTIMATES FOR FIGURES**

**Severe mental distress (≥7 GHQ-12), ages 16-34. BHPS/UKHLS, 1991-2020.**

|  | **16-24, M** | | **16-24, F** | | **25-34, M** | | **25-34, F** | |
| --- | --- | --- | --- | --- | --- | --- | --- | --- |
| **Year** | % | 95%CI | % | 95%CI | % | 95%CI | % | 95%CI |
|  |  |  |  |  |  |  |  |  |
| 1991 | 3.1 | 2.0-4.8 | 7.2 | 5.4-9.6 | 6.4 | 5.0-8.2 | 8.7 | 7.2-10.6 |
| 1992 | 3.7 | 2.6-5.3 | 7.9 | 5.9-10.4 | 6.8 | 5.2-8.9 | 10.3 | 8.4-12.4 |
| 1993 | 5.6 | 4.1-7.7 | 8.6 | 6.5-11.1 | 7.3 | 5.4-9.8 | 12.0 | 10.1-14.2 |
| 1994 | 4.9 | 3.5-7.0 | 10.5 | 8.1-13.5 | 7.8 | 5.9-10.2 | 13.1 | 11.0-15.5 |
| 1995 | 4.4 | 3.0-6.3 | 10.6 | 8.6-13.0 | 7.6 | 5.6-10.2 | 13.0 | 10.9-15.4 |
| 1996 | 5.3 | 3.6-7.7 | 10.7 | 8.2-13.8 | 9.6 | 7.5-12.2 | 11.8 | 9.5-14.5 |
| 1997 | 5.1 | 3.5-7.4 | 10.6 | 8.5-13.1 | 7.7 | 6.2-9.5 | 12.2 | 10.3-14.4 |
| 1998 | 5.9 | 4.2-8.1 | 11.5 | 9.2-14.2 | 6.6 | 4.9-8.8 | 11.8 | 9.9-14.0 |
| 1999 | 4.8 | 3.4-6.8 | 9.4 | 7.6-11.6 | 6.1 | 4.7-8.0 | 12.2 | 10.1-14.7 |
| 2000 | 5.5 | 4.1-7.5 | 12.7 | 10.5-15.3 | 6.3 | 5.0-7.9 | 13.4 | 11.3-15.7 |
| 2001 | 4.4 | 3.1-6.0 | 8.8 | 7.0-10.9 | 6.8 | 5.2-8.8 | 11.6 | 9.6-14.1 |
| 2002 | 6.7 | 4.9-9.1 | 12.6 | 10.1-15.7 | 5.4 | 3.9-7.5 | 12.8 | 10.2-15.9 |
| 2003 | 5.9 | 4.3-7.9 | 9.2 | 7.1-11.8 | 7.5 | 5.8-9.7 | 12.5 | 10.5-14.8 |
| 2004 | 4.6 | 3.2-6.7 | 13.2 | 10.5-16.6 | 6.8 | 4.9-9.3 | 9.4 | 7.5-11.7 |
| 2005 | 7.1 | 5.0-9.8 | 14.4 | 11.6-17.8 | 7.1 | 5.3-9.5 | 12.3 | 10.0-15.0 |
| 2006 | 4.6 | 3.2-6.7 | 12.3 | 9.6-15.7 | 6.6 | 4.8-9.0 | 11.1 | 8.9-13.8 |
| 2007 | 5.7 | 4.1-8.1 | 10.6 | 8.3-13.3 | 6.2 | 4.4-8.6 | 9.8 | 7.8-12.1 |
| 2008 | 5.9 | 4.1-8.3 | 12.4 | 9.7-15.7 | 7.2 | 5.3-9.8 | 10.4 | 8.1-13.3 |
| 2009 | 5.5 | 4.4-7.0 | 9.2 | 7.7-10.9 | 7.7 | 6.3-9.3 | 10.2 | 8.8-11.7 |
| 2010 | 5.6 | 4.5-6.8 | 9.3 | 8.1-10.6 | 7.4 | 6.2-8.7 | 10.0 | 8.9-11.3 |
| 2011 | 4.9 | 4.0-6.0 | 10.0 | 8.8-11.3 | 7.9 | 6.7-9.3 | 11.8 | 10.5-13.2 |
| 2012 | 6.9 | 5.8-8.3 | 13.1 | 11.5-14.8 | 7.7 | 6.4-9.1 | 14.5 | 13.1-16.1 |
| 2013 | 6.7 | 5.5-8.1 | 12.4 | 10.9-14.1 | 8.9 | 7.5-10.6 | 14.0 | 12.5-15.7 |
| 2014 | 6.4 | 5.2-7.9 | 13.1 | 11.3-15.1 | 8.0 | 6.6-9.7 | 11.0 | 9.7-12.6 |
| 2015 | 8.3 | 6.9-9.9 | 12.8 | 11.2-14.6 | 9.8 | 8.1-11.8 | 12.5 | 10.9-14.4 |
| 2016 | 8.5 | 7.0-10.3 | 13.2 | 11.4-15.1 | 9.2 | 7.6-11.2 | 13.0 | 11.3-14.9 |
| 2017 | 9.1 | 7.4-11.2 | 14.5 | 12.6-16.5 | 11.1 | 9.1-13.5 | 14.9 | 13.0-17.1 |
| 2018 | 10.6 | 8.7-12.8 | 16.3 | 14.0-18.8 | 12.9 | 10.5-15.9 | 15.3 | 13.1-17.8 |
|  |  |  |  |  |  |  |  |  |
| W10 (2018-19) | 10.7 | 8.7-13.2 | 17.5 | 15.0-20.4 | 13.6 | 11.0-16.8 | 15.4 | 12.9-18.3 |
| April 2020 | 15.9 | 10.1-24.1 | 31.7 | 26.2-37.7 | 19.1 | 13.4-26.5 | 26.3 | 21.7-31.4 |
| May 2020 | 15.8 | 10.9-22.3 | 31.4 | 24.2-39.8 | 21.2 | 15.0-29.2 | 21.3 | 16.7-26.7 |
| June 2020 | 10.8 | 6.0-18.7 | 31.9 | 23.9-41.0 | 24.4 | 17.8-32.5 | 23.5 | 17.3-31.2 |
| July 2020 | 11.1 | 5.2-22.2 | 22.0 | 15.0-31.1 | 21.0 | 14.3-29.6 | 15.0 | 11.1-19.9 |
| Sept 2020 | 12.8 | 6.3-24.1 | 18.9 | 12.4-27.8 | 15.1 | 10.2-21.9 | 15.2 | 11.1-20.5 |
|  |  |  |  |  |  |  |  |  |

**SUPPLEMENTARY TABLE 5**

**Early changes during the COVID pandemic in mental distress, ages 16-34. Great Britain, BHPS/UKHLS. 2018-2020.**

**Comparing Wave 10 (2018-19) and April 2020**

|  | **16-24, M** | | | | **16-24, F** | | | | **25-34, M** | | | | **25-34, F** | | | |
| --- | --- | --- | --- | --- | --- | --- | --- | --- | --- | --- | --- | --- | --- | --- | --- | --- |
| **Variable** | UW 10 | CW 1 | Change | 95%CI | UW 10 | CW 1 | Change | 95%CI | UW 10 | CW 1 | Change | 95%CI | UW 10 | CW 1 | Change | 95%CI |
|  | 2018-19 | 04/20 |  |  | 2018-19 | 04/20 |  |  | 2018-19 | 04/20 |  |  | 2018-19 | 04/20 |  |  |
|  |  |  |  |  |  |  |  |  |  |  |  |  |  |  |  |  |
| Score (mean) | 11.0 | 13.1 | **1.73** | **0.62; 2.84** | 13.1 | 16.1 | **2.32** | **1.60; 3.04** | 11.7 | 13.2 | **1.19** | **0.39; 1.99** | 12.6 | 15.0 | **2.85** | **2.16; 3.53** |
|  |  |  |  |  |  |  |  |  |  |  |  |  |  |  |  |  |
| ≥ 4 (%) | 20.2 | 32.0 | **1.61** | **1.20-2.16** | 32.1 | 55.3 | **1.68** | **1.47-1.91** | 21.8 | 35.9 | **1.55** | **1.25-1.92** | 24.7 | 43.9 | **1.80** | **1.56-2.08** |
| ≥ 7 (%) | 10.7 | 15.9 | 1.42 | 0.86-2.34 | 17.5 | 31.7 | **1.70** | **1.39-2.08** | 13.6 | 19.1 | 1.26 | 0.89-1.77 | 15.4 | 26.3 | **1.81** | **1.47-2.23** |
|  |  |  |  |  |  |  |  |  |  |  |  |  |  |  |  |  |
| Concentrate (%) | 15.9 | 37.2 | **2.58** | **1.97-3.38** | 26.1 | 52.6 | **1.98** | **1.70-2.30** | 18.0 | 38.0 | **1.99** | **1.61-2.47** | 23.4 | 39.2 | **1.71** | **1.46-2.01** |
| Sleep loss (%) | 10.6 | 21.2 | **1.98** | **1.34-2.93** | 23.4 | 37.1 | **1.52** | **1.28-1.80** | 18.1 | 22.6 | 1.20 | 0.91-1.58 | 21.6 | 37.7 | **1.83** | **1.57-2.14** |
| Useful (%) | 17.0 | 29.0 | **1.64** | **1.15-2.34** | 23.9 | 43.8 | **1.80** | **1.53-2.12** | 17.3 | 26.1 | **1.54** | **1.19-2.00** | 16.9 | 35.1 | **2.09** | **1.71-2.56** |
| Decisions (%) | 9.7 | 19.0 | 1.65 | 0.95-2.85 | 14.1 | 26.3 | **1.77** | **1.42-2.20** | 12.5 | 15.9 | 1.37 | 0.99-1.89 | 10.9 | 19.7 | **2.01** | **1.58-2.56** |
| Under strain (%) | 20.4 | 26.7 | 1.13 | 0.81-1.57 | 32.8 | 38.9 | 1.14 | 0.98-1.33 | 24.0 | 33.0 | 1.23 | 0.99-1.52 | 30.8 | 41.1 | **1.37** | **1.21-1.55** |
| Overcoming (%) | 16.6 | 22.1 | 1.29 | 0.88-1.87 | 24.9 | 29.1 | 1.10 | 0.91-1.34 | 16.9 | 19.6 | 1.13 | 0.86-1.49 | 18.5 | 25.1 | **1.46** | **1.21-1.77** |
| Enjoying (%) | 14.7 | 40.1 | **3.08** | **2.37-4.01** | 19.3 | 50.4 | **2.56** | **2.15-3.06** | 16.9 | 46.0 | **2.62** | **2.08-3.32** | 18.5 | 53.6 | **2.91** | **2.51-3.37** |
| Problems (%) | 11.9 | 18.6 | **1.58** | **1.00-2.47** | 16.2 | 30.2 | **1.76** | **1.43-2.18** | 11.7 | 19.5 | **1.57** | **1.09-2.26** | 13.6 | 20.9 | **1.65** | **1.31-2.08** |
| Depressed (%) | 21.1 | 27.1 | 1.34 | 0.98-1.83 | 31.6 | 54.7 | **1.68** | **1.48-1.91** | 23.3 | 34.5 | **1.47** | **1.16-1.85** | 26.3 | 41.4 | **1.62** | **1.40-1.87** |
| Confidence (%) | 18.6 | 18.8 | 0.98 | 0.67-1.41 | 31.7 | 45.4 | **1.38** | **1.19-1.60** | 20.1 | 22.0 | 1.03 | 0.78-1.36 | 27.2 | 34.7 | **1.29** | **1.10-1.50** |
| Self-worth (%) | 12.7 | 16.7 | 1.38 | 0.85-2.23 | 20.1 | 26.6 | 1.22 | 0.99-1.51 | 13.0 | 13.1 | 1.22 | 0.81-1.84 | 14.6 | 18.9 | **1.32** | **1.03-1.69** |
| Happiness (%) | 13.7 | 23.6 | **2.01** | **1.42-2.84** | 19.8 | 34.8 | **1.70** | **1.39-2.09** | 17.3 | 26.7 | **1.44** | **1.10-1.88** | 18.2 | 35.2 | **1.97** | **1.65-2.34** |
|  |  |  |  |  |  |  |  |  |  |  |  |  |  |  |  |  |

Change coefficients are subject-specific linear betas and prevalence ratios from random-effects models, weighted with clustered errors.

Bolded estimates are significant at the < .05 level.

UW 10 = UKHLS wave 10 participants surveyed between January 2018 and December 2019.

CW 1 = COVID wave 1 participants surveyed between April 24^th^ and 30^th^ 2020.

**SUPPLEMENTARY TABLE 6**

**Early changes during the COVID pandemic, ages 16-34. Great Britain, BHPS/UKHLS. 2020.**

**Comparing April 2020 and July-Sept 2020**

|  | **16-24, M** | | | | **16-24, F** | | | | **25-34, M** | | | | **25-34, F** | | | |
| --- | --- | --- | --- | --- | --- | --- | --- | --- | --- | --- | --- | --- | --- | --- | --- | --- |
| **Variable** | CW 1 | CW 4-5 | Change | 95%CI | CW 1 | CW 4-5 | Change | 95%CI | CW 1 | CW 4-5 | Change | 95%CI | CW 1 | CW 4-5 | Change | 95%CI |
|  | 04/20 | 08-09/20 |  |  | 04/20 | 08-09/20 |  |  | 04/20 | 08-09/20 |  |  | 04/20 | 08-09/20 |  |  |
|  |  |  |  |  |  |  |  |  |  |  |  |  |  |  |  |  |
| Score (mean) | 13.1 | 11.8 | -0.25 | -0.94; 0.43 | 16.1 | 14.1 | **-2.19** | **-2.98; -1.40** | 13.2 | 13.3 | 0.12 | -0.77; 1.00 | 15.0 | 13.3 | **-1.91** | **-2.56; -1.26** |
|  |  |  |  |  |  |  |  |  |  |  |  |  |  |  |  |  |
| ≥ 4 (%) | 32.0 | 17.3 | **0.70** | **0.51-0.95** | 55.3 | 31.9 | **0.54** | **0.45-0.65** | 35.9 | 28.0 | **0.79** | **0.65-0.97** | 43.9 | 25.3 | **0.57** | **0.48-0.67** |
| ≥ 7 (%) | 15.9 | 11.9 | 0.96 | 0.62-1.50 | 31.7 | 20.5 | **0.59** | **0.45-0.77** | 19.1 | 18.1 | 1.01 | 0.74-1.40 | 26.3 | 15.1 | **0.57** | **0.46-0.71** |
|  |  |  |  |  |  |  |  |  |  |  |  |  |  |  |  |  |
| Concentrate (%) | 37.2 | 14.5 | **0.43** | **0.27-0.68** | 52.6 | 29.5 | **0.51** | **0.42-0.62** | 38.0 | 21.7 | **0.57** | **0.43-0.76** | 39.2 | 21.3 | **0.54** | **0.45-0.65** |
| Sleep loss (%) | 21.2 | 8.8 | **0.64** | **0.42-0.98** | 37.1 | 23.8 | **0.61** | **0.46-0.80** | 22.6 | 23.7 | 0.95 | 0.70-1.27 | 37.7 | 21.7 | **0.58** | **0.49-0.69** |
| Useful (%) | 29.0 | 17.2 | **0.67** | **0.46-0.97** | 43.8 | 28.0 | **0.61** | **0.49-0.75** | 26.1 | 19.4 | 0.75 | 0.52-1.07 | 35.1 | 19.3 | **0.54** | **0.45-0.65** |
| Decisions (%) | 19.0 | 11.8 | 0.98 | 0.57-1.69 | 26.3 | 18.8 | **0.74** | **0.56-0.98** | 15.9 | 13.1 | 0.88 | 0.61-1.27 | 19.7 | 14.8 | **0.76** | **0.61-0.93** |
| Under strain (%) | 26.7 | 17.3 | 0.87 | 0.60-1.25 | 38.9 | 31.4 | **0.78** | **0.63-0.96** | 33.0 | 33.1 | 1.02 | 0.81-1.28 | 41.1 | 30.3 | **0.75** | **0.64-0.87** |
| Overcoming (%) | 22.1 | 18.3 | 0.82 | 0.61-1.12 | 29.1 | 26.6 | 0.82 | 0.66-1.03 | 19.6 | 24.7 | 1.19 | 0.86-1.65 | 25.1 | 19.0 | **0.75** | **0.61-0.92** |
| Enjoying (%) | 40.1 | 17.8 | **0.47** | **0.32-0.70** | 50.4 | 24.8 | **0.45** | **0.37-0.55** | 46.0 | 24.3 | **0.52** | **0.40-0.69** | 53.6 | 24.0 | **0.44** | **0.38-0.51** |
| Problems (%) | 18.6 | 14.5 | 0.90 | 0.62-1.31 | 30.2 | 19.4 | **0.55** | **0.40-0.75** | 19.5 | 17.0 | 0.96 | 0.68-1.36 | 20.9 | 15.7 | **0.72** | **0.59-0.88** |
| Depressed (%) | 27.1 | 18.0 | 0.80 | 0.58-1.11 | 54.7 | 32.0 | **0.55** | **0.46-0.65** | 34.5 | 27.5 | **0.74** | **0.56-0.98** | 41.4 | 26.9 | **0.63** | **0.54-0.73** |
| Confidence (%) | 18.8 | 14.6 | 0.95 | 0.69-1.31 | 45.4 | 31.0 | **0.63** | **0.52-0.76** | 22.0 | 23.7 | 0.97 | 0.74-1.27 | 34.7 | 25.1 | **0.70** | **0.59-0.84** |
| Self-worth (%) | 16.7 | 11.7 | 0.75 | 0.50-1.14 | 26.6 | 20.0 | 0.77 | 0.59-1.02 | 13.1 | 15.4 | 1.08 | 0.72-1.63 | 18.9 | 16.1 | **0.79** | **0.63-0.99** |
| Happiness (%) | 23.6 | 13.5 | 0.67 | 0.43-1.04 | 34.8 | 20.6 | **0.57** | **0.43-0.75** | 26.7 | 24.4 | 0.94 | 0.73-1.22 | 35.2 | 20.7 | **0.58** | **0.48-0.70** |
|  |  |  |  |  |  |  |  |  |  |  |  |  |  |  |  |  |

Change coefficients are subject-specific linear betas and prevalence ratios from random-effects models, weighted with clustered errors.

Bolded estimates are significant at the < .05 level.

CW 1 = COVID wave 1 participants surveyed between April 24^th^ and 30^th^ 2020.

CW 4-5 = COVID waves 4-5 participants surveyed between July 24^th^ and October 1^st^ 2020.

**SUPPLEMENTARY FIGURE 1**

**Long-term trends in clinical thresholds based on GHQ caseness score (%), ages 16-34. Great Britain, 1999-2018**

**SUPPLEMENTARY FIGURE 2**

**Recent changes in clinical thresholds based on GHQ caseness score (%), ages 16-34. Great Britain, 2018-2020**
